# Supplementary material for: Pathophysiological Significance of Neutrophilic Transfer RNA-Derived Small RNAs in Asymptomatic Moyamoya Disease
Source: Cells. 2021 May 1;10(5):1086. doi: 10.3390/cells10051086 (PMC8147334; doi:10.3390/cells10051086)

2D Structure

54 69

8mer-1a

5 ' - c c g a t t g C A C C A G G G a - 3 ' **utr**

3 ' - u g a u c u g G U G G U C C C u - 5 ' **tRF**

16 15 14 13 7 6 5 4 3 2

3' Pairing Seed

Local AU

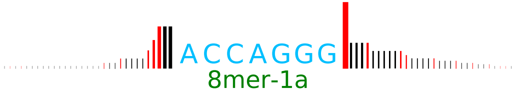

Position

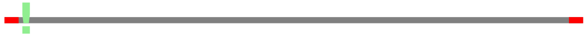

Supplement: Supplementary file 1 [file cells-10-01086-s001.zip › cells-1149161 3rd supplementary materials/Data Supplements/Data Supplement V.zip!/Data supplement 6-mRNAs targets of tsRNAs/tsRNA-Glu-CTC-003/Diagrams/tRF-Glu-CTC-003_vs_ENST00000267205(RHOF).pdf]
